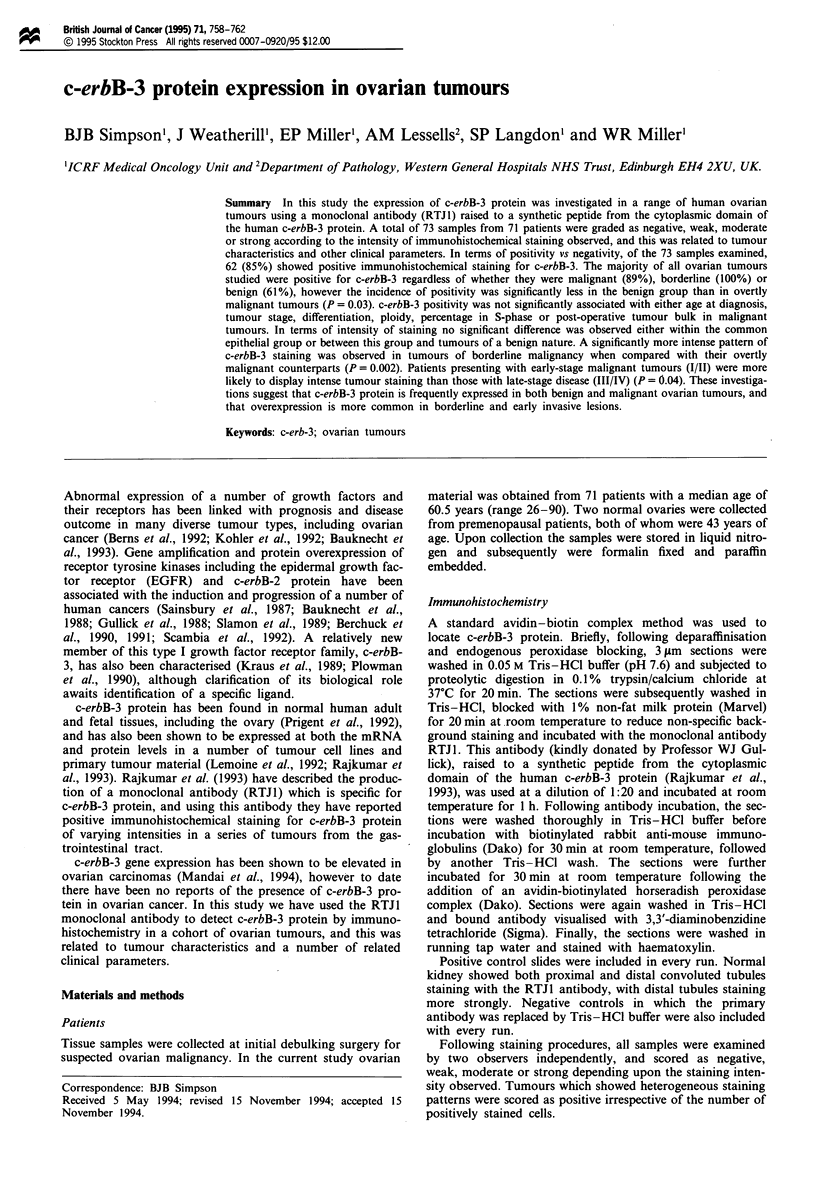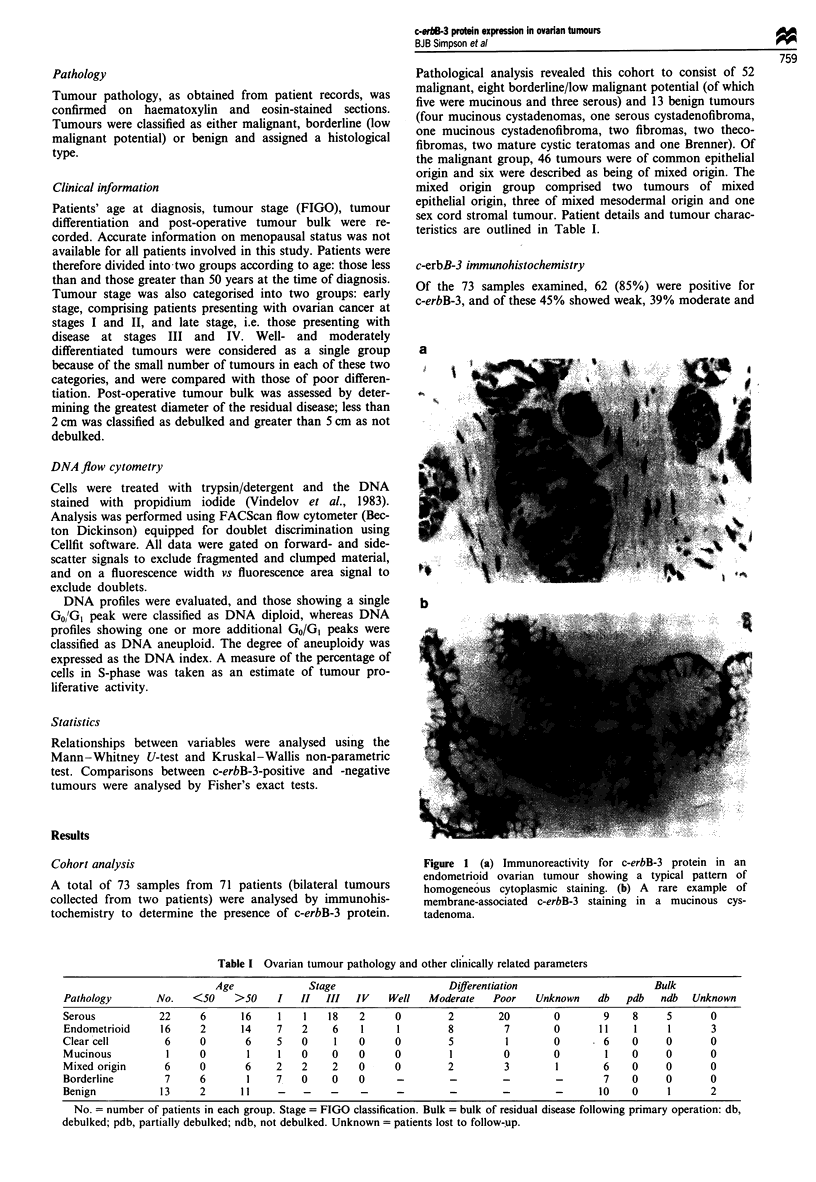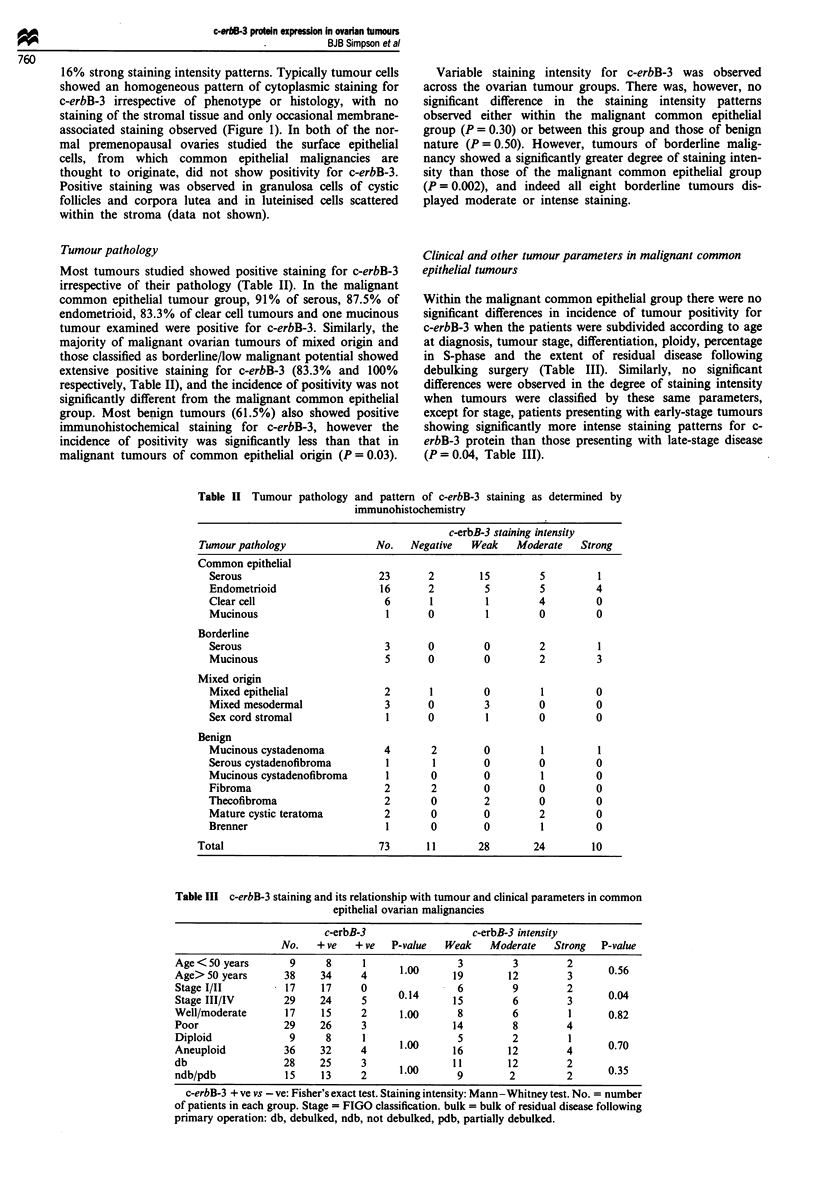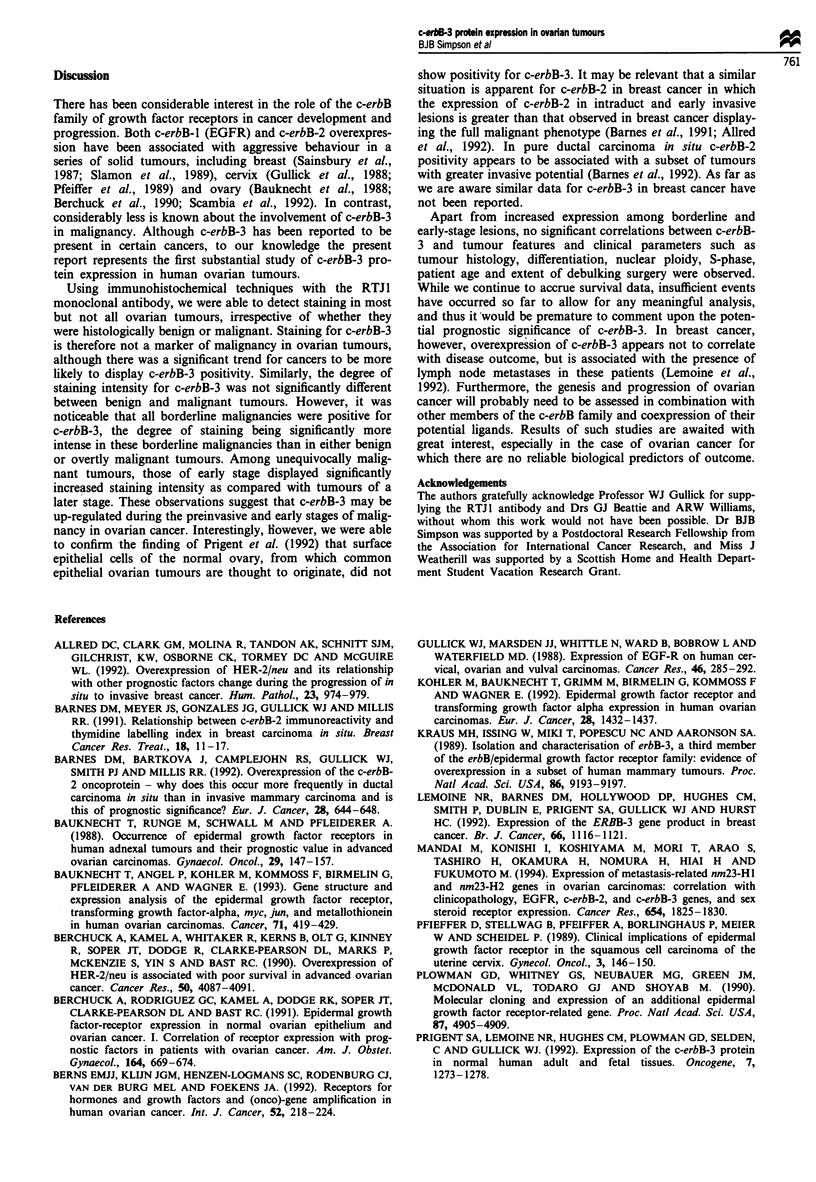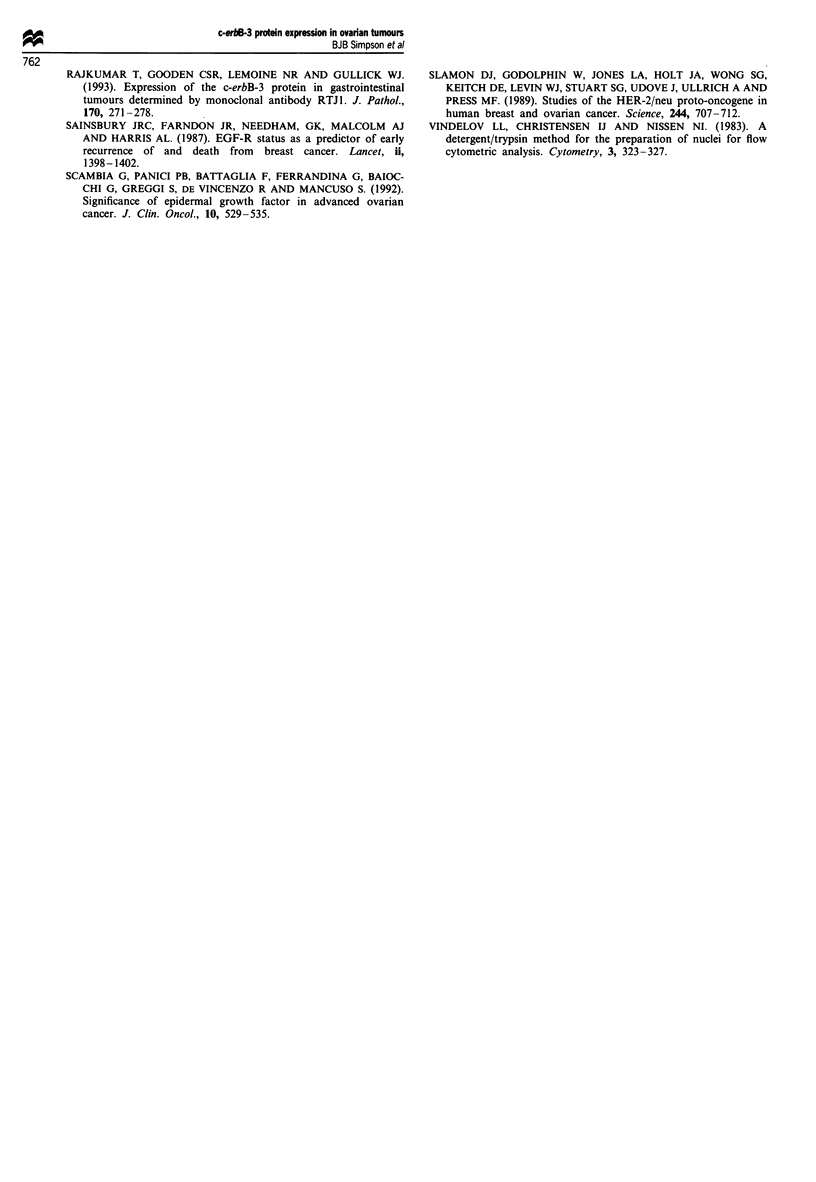# c-erbB-3 protein expression in ovarian tumours.

**DOI:** 10.1038/bjc.1995.147

**Published:** 1995-04

**Authors:** B. J. Simpson, J. Weatherill, E. P. Miller, A. M. Lessells, S. P. Langdon, W. R. Miller

**Affiliations:** ICRF Medical Oncology Unit, Western General Hospitals NHS Trust, Edinburgh, UK.

## Abstract

**Images:**


British Journal of Cancer (1995) 71, 758-762
© 1995 Stockton Press All rights reserved 0007-0920/95 $12.00

### c-*erb*B-3 protein expression in ovarian tumours

BJB Simpson[1], J Weatherill[1], EP Miller[1], AM Lessells[2], SP Langdon[1] and WR Miller[1]

[1]*ICRF Medical Oncology Unit and* [2]*Department of Pathology, Western General Hospitals NHS Trust, Edinburgh EH4 2XU, UK.*

**Summary** In this study the expression of c-*erb*B-3 protein was investigated in a range of human ovarian tumours using a monoclonal antibody (RTJ1) raised to a synthetic peptide from the cytoplasmic domain of the human c-*erb*B-3 protein. A total of 73 samples from 71 patients were graded as negative, weak, moderate or strong according to the intensity of immunohistochemical staining observed, and this was related to tumour characteristics and other clinical parameters. In terms of positivity *vs* negativity, of the 73 samples examined, 62 (85%) showed positive immunohistochemical staining for c-*erb*B-3. The majority of all ovarian tumours studied were positive for c-*erb*B-3 regardless of whether they were malignant (89%), borderline (100%) or benign (61%), however the incidence of positivity was significantly less in the benign group than in overtly malignant tumours ($P = 0.03$). c-*erb*B-3 positivity was not significantly associated with either age at diagnosis, tumour stage, differentiation, ploidy, percentage in S-phase or post-operative tumour bulk in malignant tumours. In terms of intensity of staining no significant difference was observed either within the common epithelial group or between this group and tumours of a benign nature. A significantly more intense pattern of c-*erb*B-3 staining was observed in tumours of borderline malignancy when compared with their overtly malignant counterparts ($P = 0.002$). Patients presenting with early-stage malignant tumours (I/II) were more likely to display intense tumour staining than those with late-stage disease (III/IV) ($P = 0.04$). These investigations suggest that c-*erb*B-3 protein is frequently expressed in both benign and malignant ovarian tumours, and that overexpression is more common in borderline and early invasive lesions.

**Keywords**: c-*erb*-3; ovarian tumours

Abnormal expression of a number of growth factors and their receptors has been linked with prognosis and disease outcome in many diverse tumour types, including ovarian cancer (Berns *et al.*, 1992; Kohler *et al.*, 1992; Bauknecht *et al.*, 1993). Gene amplification and protein overexpression of receptor tyrosine kinases including the epidermal growth factor receptor (EGFR) and c-*erb*B-2 protein have been associated with the induction and progression of a number of human cancers (Sainsbury *et al.*, 1987; Bauknecht *et al.*, 1988; Gullick *et al.*, 1988; Slamon *et al.*, 1989; Berchuck *et al.*, 1990, 1991; Scambia *et al.*, 1992). A relatively new member of this type I growth factor receptor family, c-*erb*B-3, has also been characterised (Kraus *et al.*, 1989; Plowman *et al.*, 1990), although clarification of its biological role awaits identification of a specific ligand.

c-*erb*B-3 protein has been found in normal human adult and fetal tissues, including the ovary (Prigent *et al.*, 1992), and has also been shown to be expressed at both the mRNA and protein levels in a number of tumour cell lines and primary tumour material (Lemoine *et al.*, 1992; Rajkumar *et al.*, 1993). Rajkumar *et al.* (1993) have described the production of a monoclonal antibody (RTJ1) which is specific for c-*erb*B-3 protein, and using this antibody they have reported positive immunohistochemical staining for c-*erb*B-3 protein of varying intensities in a series of tumours from the gastrointestinal tract.

c-*erb*B-3 gene expression has been shown to be elevated in ovarian carcinomas (Mandai *et al.*, 1994), however to date there have been no reports of the presence of c-*erb*B-3 protein in ovarian cancer. In this study we have used the RTJ1 monoclonal antibody to detect c-*erb*B-3 protein by immunohistochemistry in a cohort of ovarian tumours, and this was related to tumour characteristics and a number of related clinical parameters.

### Materials and methods

### *Patients*

Tissue samples were collected at initial debulking surgery for suspected ovarian malignancy. In the current study ovarian material was obtained from 71 patients with a median age of 60.5 years (range 26-90). Two normal ovaries were collected from premenopausal patients, both of whom were 43 years of age. Upon collection the samples were stored in liquid nitrogen and subsequently were formalin fixed and paraffin embedded.

### *Immunohistochemistry*

A standard avidin-biotin complex method was used to locate c-*erb*B-3 protein. Briefly, following deparaffinisation and endogenous peroxidase blocking, 3 μm sections were washed in 0.05 M Tris-HCl buffer (pH 7.6) and subjected to proteolytic digestion in 0.1% trypsin/calcium chloride at 37°C for 20 min. The sections were subsequently washed in Tris-HCl, blocked with 1% non-fat milk protein (Marvel) for 20 min at room temperature to reduce non-specific background staining and incubated with the monoclonal antibody RTJ1. This antibody (kindly donated by Professor WJ Gullick), raised to a synthetic peptide from the cytoplasmic domain of the human c-*erb*B-3 protein (Rajkumar *et al.*, 1993), was used at a dilution of 1:20 and incubated at room temperature for 1 h. Following antibody incubation, the sections were washed thoroughly in Tris-HCl buffer before incubation with biotinylated rabbit anti-mouse immunoglobulins (Dako) for 30 min at room temperature, followed by another Tris-HCl wash. The sections were further incubated for 30 min at room temperature following the addition of an avidin-biotinylated horseradish peroxidase complex (Dako). Sections were again washed in Tris-HCl and bound antibody visualised with 3,3′-diaminobenzidine tetrachloride (Sigma). Finally, the sections were washed in running tap water and stained with haematoxylin.

Positive control slides were included in every run. Normal kidney showed both proximal and distal convoluted tubules staining with the RTJ1 antibody, with distal tubules staining more strongly. Negative controls in which the primary antibody was replaced by Tris-HCl buffer were also included with every run.

Following staining procedures, all samples were examined by two observers independently, and scored as negative, weak, moderate or strong depending upon the staining intensity observed. Tumours which showed heterogeneous staining patterns were scored as positive irrespective of the number of positively stained cells.

Correspondence: BJB Simpson
Received 5 May 1994; revised 15 November 1994; accepted 15 November 1994.

### *Pathology*

Tumour pathology, as obtained from patient records, was confirmed on haematoxylin and eosin-stained sections. Tumours were classified as either malignant, borderline (low malignant potential) or benign and assigned a histological type.

### *Clinical information*

Patients' age at diagnosis, tumour stage (FIGO), tumour differentiation and post-operative tumour bulk were recorded. Accurate information on menopausal status was not available for all patients involved in this study. Patients were therefore divided into two groups according to age: those less than and those greater than 50 years at the time of diagnosis. Tumour stage was also categorised into two groups: early stage, comprising patients presenting with ovarian cancer at stages I and II, and late stage, i.e. those presenting with disease at stages III and IV. Well- and moderately differentiated tumours were considered as a single group because of the small number of tumours in each of these two categories, and were compared with those of poor differentiation. Post-operative tumour bulk was assessed by determining the greatest diameter of the residual disease; less than 2 cm was classified as debulked and greater than 5 cm as not debulked.

### *DNA flow cytometry*

Cells were treated with trypsin/detergent and the DNA stained with propidium iodide (Vindelov *et al.*, 1983). Analysis was performed using FACScan flow cytometer (Becton Dickinson) equipped for doublet discrimination using Cellfit software. All data were gated on forward- and side-scatter signals to exclude fragmented and clumped material, and on a fluorescence width *vs* fluorescence area signal to exclude doublets.

DNA profiles were evaluated, and those showing a single $G_0/G_1$ peak were classified as DNA diploid, whereas DNA profiles showing one or more additional $G_0/G_1$ peaks were classified as DNA aneuploid. The degree of aneuploidy was expressed as the DNA index. A measure of the percentage of cells in S-phase was taken as an estimate of tumour proliferative activity.

### *Statistics*

Relationships between variables were analysed using the Mann–Whitney *U*-test and Kruskal–Wallis non-parametric test. Comparisons between c-*erb*B-3-positive and -negative tumours were analysed by Fisher's exact tests.

### Results

### *Cohort analysis*

A total of 73 samples from 71 patients (bilateral tumours collected from two patients) were analysed by immunohistochemistry to determine the presence of c-*erb*B-3 protein. Pathological analysis revealed this cohort to consist of 52 malignant, eight borderline/low malignant potential (of which five were mucinous and three serous) and 13 benign tumours (four mucinous cystadenomas, one serous cystadenofibroma, one mucinous cystadenofibroma, two fibromas, two thecofibromas, two mature cystic teratomas and one Brenner). Of the malignant group, 46 tumours were of common epithelial origin and six were described as being of mixed origin. The mixed origin group comprised two tumours of mixed epithelial origin, three of mixed mesodermal origin and one sex cord stromal tumour. Patient details and tumour characteristics are outlined in Table I.

### c-erb*B-3 immunohistochemistry*

Of the 73 samples examined, 62 (85%) were positive for c-*erb*B-3, and of these 45% showed weak, 39% moderate and

**Figure 1** (**a**) Immunoreactivity for c-*erb*B-3 protein in an endometrioid ovarian tumour showing a typical pattern of homogeneous cytoplasmic staining. (**b**) A rare example of membrane-associated c-*erb*B-3 staining in a mucinous cystadenoma.

**Table I** Ovarian tumour pathology and other clinically related parameters

| | | *Age* | | *Stage* | | | | *Differentiation* | | | | *Bulk* | | | |
|---|---|---|---|---|---|---|---|---|---|---|---|---|---|---|---|
| *Pathology* | *No.* | *<50* | *>50* | *I* | *II* | *III* | *IV* | *Well* | *Moderate* | *Poor* | *Unknown* | *db* | *pdb* | *ndb* | *Unknown* |
| Serous | 22 | 6 | 16 | 1 | 1 | 18 | 2 | 0 | 2 | 20 | 0 | 9 | 8 | 5 | 0 |
| Endometrioid | 16 | 2 | 14 | 7 | 2 | 6 | 1 | 1 | 8 | 7 | 0 | 11 | 1 | 1 | 3 |
| Clear cell | 6 | 0 | 6 | 5 | 0 | 1 | 0 | 0 | 5 | 1 | 0 | 6 | 0 | 0 | 0 |
| Mucinous | 1 | 0 | 1 | 1 | 0 | 0 | 0 | 0 | 1 | 0 | 0 | 1 | 0 | 0 | 0 |
| Mixed origin | 6 | 0 | 6 | 2 | 2 | 2 | 0 | 0 | 2 | 3 | 1 | 6 | 0 | 0 | 0 |
| Borderline | 7 | 6 | 1 | 7 | 0 | 0 | 0 | – | – | – | – | 7 | 0 | 0 | 0 |
| Benign | 13 | 2 | 11 | – | – | – | – | – | – | – | – | 10 | 0 | 1 | 2 |

No. = number of patients in each group. Stage = FIGO classification. Bulk = bulk of residual disease following primary operation: db, debulked; pdb, partially debulked; ndb, not debulked. Unknown = patients lost to follow-up.

16% strong staining intensity patterns. Typically tumour cells showed an homogeneous pattern of cytoplasmic staining for c-*erb*B-3 irrespective of phenotype or histology, with no staining of the stromal tissue and only occasional membrane-associated staining observed (Figure 1). In both of the normal premenopausal ovaries studied the surface epithelial cells, from which common epithelial malignancies are thought to originate, did not show positivity for c-*erb*B-3. Positive staining was observed in granulosa cells of cystic follicles and corpora lutea and in luteinised cells scattered within the stroma (data not shown).

### *Tumour pathology*

Most tumours studied showed positive staining for c-*erb*B-3 irrespective of their pathology (Table II). In the malignant common epithelial tumour group, 91% of serous, 87.5% of endometrioid, 83.3% of clear cell tumours and one mucinous tumour examined were positive for c-*erb*B-3. Similarly, the majority of malignant ovarian tumours of mixed origin and those classified as borderline/low malignant potential showed extensive positive staining for c-*erb*B-3 (83.3% and 100% respectively, Table II), and the incidence of positivity was not significantly different from the malignant common epithelial group. Most benign tumours (61.5%) also showed positive immunohistochemical staining for c-*erb*B-3, however the incidence of positivity was significantly less than that in malignant tumours of common epithelial origin ($P = 0.03$).

Variable staining intensity for c-*erb*B-3 was observed across the ovarian tumour groups. There was, however, no significant difference in the staining intensity patterns observed either within the malignant common epithelial group ($P = 0.30$) or between this group and those of benign nature ($P = 0.50$). However, tumours of borderline malignancy showed a significantly greater degree of staining intensity than those of the malignant common epithelial group ($P = 0.002$), and indeed all eight borderline tumours displayed moderate or intense staining.

### *Clinical and other tumour parameters in malignant common epithelial tumours*

Within the malignant common epithelial group there were no significant differences in incidence of tumour positivity for c-*erb*B-3 when the patients were subdivided according to age at diagnosis, tumour stage, differentiation, ploidy, percentage in S-phase and the extent of residual disease following debulking surgery (Table III). Similarly, no significant differences were observed in the degree of staining intensity when tumours were classified by these same parameters, except for stage, patients presenting with early-stage tumours showing significantly more intense staining patterns for c-*erb*B-3 protein than those presenting with late-stage disease ($P = 0.04$, Table III).

**Table II** Tumour pathology and pattern of c-*erb*B-3 staining as determined by immunohistochemistry

| | | *c-*erb*B-3 staining intensity* | | | |
|---|---|---|---|---|---|
| *Tumour pathology* | *No.* | *Negative* | *Weak* | *Moderate* | *Strong* |
| Common epithelial | | | | | |
| Serous | 23 | 2 | 15 | 5 | 1 |
| Endometrioid | 16 | 2 | 5 | 5 | 4 |
| Clear cell | 6 | 1 | 1 | 4 | 0 |
| Mucinous | 1 | 0 | 1 | 0 | 0 |
| Borderline | | | | | |
| Serous | 3 | 0 | 0 | 2 | 1 |
| Mucinous | 5 | 0 | 0 | 2 | 3 |
| Mixed origin | | | | | |
| Mixed epithelial | 2 | 1 | 0 | 1 | 0 |
| Mixed mesodermal | 3 | 0 | 3 | 0 | 0 |
| Sex cord stromal | 1 | 0 | 1 | 0 | 0 |
| Benign | | | | | |
| Mucinous cystadenoma | 4 | 2 | 0 | 1 | 1 |
| Serous cystadenofibroma | 1 | 1 | 0 | 0 | 0 |
| Mucinous cystadenofibroma | 1 | 0 | 0 | 1 | 0 |
| Fibroma | 2 | 2 | 0 | 0 | 0 |
| Thecofibroma | 2 | 0 | 2 | 0 | 0 |
| Mature cystic teratoma | 2 | 0 | 0 | 2 | 0 |
| Brenner | 1 | 0 | 0 | 1 | 0 |
| Total | 73 | 11 | 28 | 24 | 10 |

**Table III** c-*erb*B-3 staining and its relationship with tumour and clinical parameters in common epithelial ovarian malignancies

| | | *c-*erb*B-3* | | | *c-*erb*B-3 intensity* | | | |
|---|---|---|---|---|---|---|---|---|
| | *No.* | *+ve* | *+ve* | P-*value* | *Weak* | *Moderate* | *Strong* | P-*value* |
| Age < 50 years | 9 | 8 | 1 | 1.00 | 3 | 3 | 2 | 0.56 |
| Age > 50 years | 38 | 34 | 4 | | 19 | 12 | 3 | |
| Stage I/II | 17 | 17 | 0 | 0.14 | 6 | 9 | 2 | 0.04 |
| Stage III/IV | 29 | 24 | 5 | | 15 | 6 | 3 | |
| Well/moderate | 17 | 15 | 2 | 1.00 | 8 | 6 | 1 | 0.82 |
| Poor | 29 | 26 | 3 | | 14 | 8 | 4 | |
| Diploid | 9 | 8 | 1 | 1.00 | 5 | 2 | 1 | 0.70 |
| Aneuploid | 36 | 32 | 4 | | 16 | 12 | 4 | |
| db | 28 | 25 | 3 | 1.00 | 11 | 12 | 2 | 0.35 |
| ndb/pdb | 15 | 13 | 2 | | 9 | 2 | 2 | |

c-*erb*B-3 + ve *vs* – ve: Fisher's exact test. Staining intensity: Mann–Whitney test. No. = number of patients in each group. Stage = FIGO classification. bulk = bulk of residual disease following primary operation: db, debulked, ndb, not debulked, pdb, partially debulked.

### Discussion

There has been considerable interest in the role of the c-*erb*B family of growth factor receptors in cancer development and progression. Both c-*erb*B-1 (EGFR) and c-*erb*B-2 overexpression have been associated with aggressive behaviour in a series of solid tumours, including breast (Sainsbury *et al.*, 1987; Slamon *et al.*, 1989), cervix (Gullick *et al.*, 1988; Pfeiffer *et al.*, 1989) and ovary (Bauknecht *et al.*, 1988; Berchuck *et al.*, 1990; Scambia *et al.*, 1992). In contrast, considerably less is known about the involvement of c-*erb*B-3 in malignancy. Although c-*erb*B-3 has been reported to be present in certain cancers, to our knowledge the present report represents the first substantial study of c-*erb*B-3 protein expression in human ovarian tumours.

Using immunohistochemical techniques with the RTJ1 monoclonal antibody, we were able to detect staining in most but not all ovarian tumours, irrespective of whether they were histologically benign or malignant. Staining for c-*erb*B-3 is therefore not a marker of malignancy in ovarian tumours, although there was a significant trend for cancers to be more likely to display c-*erb*B-3 positivity. Similarly, the degree of staining intensity for c-*erb*B-3 was not significantly different between benign and malignant tumours. However, it was noticeable that all borderline malignancies were positive for c-*erb*B-3, the degree of staining being significantly more intense in these borderline malignancies than in either benign or overtly malignant tumours. Among unequivocally malignant tumours, those of early stage displayed significantly increased staining intensity as compared with tumours of a later stage. These observations suggest that c-*erb*B-3 may be up-regulated during the preinvasive and early stages of malignancy in ovarian cancer. Interestingly, however, we were able to confirm the finding of Prigent *et al.* (1992) that surface epithelial cells of the normal ovary, from which common epithelial ovarian tumours are thought to originate, did not show positivity for c-*erb*B-3. It may be relevant that a similar situation is apparent for c-*erb*B-2 in breast cancer in which the expression of c-*erb*B-2 in intraduct and early invasive lesions is greater than that observed in breast cancer displaying the full malignant phenotype (Barnes *et al.*, 1991; Allred *et al.*, 1992). In pure ductal carcinoma *in situ* c-*erb*B-2 positivity appears to be associated with a subset of tumours with greater invasive potential (Barnes *et al.*, 1992). As far as we are aware similar data for c-*erb*B-3 in breast cancer have not been reported.

Apart from increased expression among borderline and early-stage lesions, no significant correlations between c-*erb*B-3 and tumour features and clinical parameters such as tumour histology, differentiation, nuclear ploidy, S-phase, patient age and extent of debulking surgery were observed. While we continue to accrue survival data, insufficient events have occurred so far to allow for any meaningful analysis, and thus it would be premature to comment upon the potential prognostic significance of c-*erb*B-3. In breast cancer, however, overexpression of c-*erb*B-3 appears not to correlate with disease outcome, but is associated with the presence of lymph node metastases in these patients (Lemoine *et al.*, 1992). Furthermore, the genesis and progression of ovarian cancer will probably need to be assessed in combination with other members of the c-*erb*B family and coexpression of their potential ligands. Results of such studies are awaited with great interest, especially in the case of ovarian cancer for which there are no reliable biological predictors of outcome.

**Acknowledgements**

The authors gratefully acknowledge Professor WJ Gullick for supplying the RTJ1 antibody and Drs GJ Beattie and ARW Williams, without whom this work would not have been possible. Dr BJB Simpson was supported by a Postdoctoral Research Fellowship from the Association for International Cancer Research, and Miss J Weatherill was supported by a Scottish Home and Health Department Student Vacation Research Grant.

### References

ALLRED DC, CLARK GM, MOLINA R, TANDON AK, SCHNITT SJM, GILCHRIST, KW, OSBORNE CK, TORMEY DC AND MCGUIRE WL. (1992). Overexpression of HER-2/*neu* and its relationship with other prognostic factors change during the progression of *in situ* to invasive breast cancer. *Hum. Pathol.*, **23**, 974–979.

BARNES DM, MEYER JS, GONZALES JG, GULLICK WJ AND MILLIS RR. (1991). Relationship between c-*erb*B-2 immunoreactivity and thymidine labelling index in breast carcinoma *in situ*. *Breast Cancer Res. Treat.*, **18**, 11–17.

BARNES DM, BARTKOVA J, CAMPLEJOHN RS, GULLICK WJ, SMITH PJ AND MILLIS RR. (1992). Overexpression of the c-*erb*B-2 oncoprotein – why does this occur more frequently in ductal carcinoma *in situ* than in invasive mammary carcinoma and is this of prognostic significance? *Eur. J. Cancer*, **28**, 644–648.

BAUKNECHT T, RUNGE M, SCHWALL M AND PFLEIDERER A. (1988). Occurrence of epidermal growth factor receptors in human adnexal tumours and their prognostic value in advanced ovarian carcinomas. *Gynaecol. Oncol.*, **29**, 147–157.

BAUKNECHT T, ANGEL P, KOHLER M, KOMMOSS F, BIRMELIN G, PFLEIDERER A AND WAGNER E. (1993). Gene structure and expression analysis of the epidermal growth factor receptor, transforming growth factor-alpha, *myc*, *jun*, and metallothionein in human ovarian carcinomas. *Cancer*, **71**, 419–429.

BERCHUCK A, KAMEL A, WHITAKER R, KERNS B, OLT G, KINNEY R, SOPER JT, DODGE R, CLARKE-PEARSON DL, MARKS P, MCKENZIE S, YIN S AND BAST RC. (1990). Overexpression of HER-2/neu is associated with poor survival in advanced ovarian cancer. *Cancer Res.*, **50**, 4087-4091.

BERCHUCK A, RODRIGUEZ GC, KAMEL A, DODGE RK, SOPER JT, CLARKE-PEARSON DL AND BAST RC. (1991). Epidermal growth factor-receptor expression in normal ovarian epithelium and ovarian cancer. I. Correlation of receptor expression with prognostic factors in patients with ovarian cancer. *Am. J. Obstet. Gynaecol.*, **164**, 669–674.

BERNS EMJJ, KLIJN JGM, HENZEN-LOGMANS SC, RODENBURG CJ, VAN DER BURG MEL AND FOEKENS JA. (1992). Receptors for hormones and growth factors and (onco)-gene amplification in human ovarian cancer. *Int. J. Cancer*, **52**, 218–224.

GULLICK WJ, MARSDEN JJ, WHITTLE N, WARD B, BOBROW L AND WATERFIELD MD. (1988). Expression of EGF-R on human cervical, ovarian and vulval carcinomas. *Cancer Res.*, **46**, 285–292.

KOHLER M, BAUKNECHT T, GRIMM M, BIRMELIN G, KOMMOSS F AND WAGNER E. (1992). Epidermal growth factor receptor and transforming growth factor alpha expression in human ovarian carcinomas. *Eur. J. Cancer*, **28**, 1432–1437.

KRAUS MH, ISSING W, MIKI T, POPESCU NC AND AARONSON SA. (1989). Isolation and characterisation of *erb*B-3, a third member of the *erb*B/epidermal growth factor receptor family: evidence of overexpression in a subset of human mammary tumours. *Proc. Natl Acad. Sci. USA*, **86**, 9193–9197.

LEMOINE NR, BARNES DM, HOLLYWOOD DP, HUGHES CM, SMITH P, DUBLIN E, PRIGENT SA, GULLICK WJ AND HURST HC. (1992). Expression of the *ERB*B-3 gene product in breast cancer. *Br. J. Cancer*, **66**, 1116–1121.

MANDAI M, KONISHI I, KOSHIYAMA M, MORI T, ARAO S, TASHIRO H, OKAMURA H, NOMURA H, HIAI H AND FUKUMOTO M. (1994). Expression of metastasis-related *nm*23-H1 and *nm*23-H2 genes in ovarian carcinomas: correlation with clinicopathology, EGFR, c-*erb*B-2, and c-*erb*B-3 genes, and sex steroid receptor expression. *Cancer Res.*, **654**, 1825–1830.

PFIEFFER D, STELLWAG B, PFEIFFER A, BORLINGHAUS P, MEIER W AND SCHEIDEL P. (1989). Clinical implications of epidermal growth factor receptor in the squamous cell carcinoma of the uterine cervix. *Gynecol. Oncol.*, **3**, 146–150.

PLOWMAN GD, WHITNEY GS, NEUBAUER MG, GREEN JM, MCDONALD VL, TODARO GJ AND SHOYAB M. (1990). Molecular cloning and expression of an additional epidermal growth factor receptor-related gene. *Proc. Natl Acad. Sci. USA*, **87**, 4905–4909.

PRIGENT SA, LEMOINE NR, HUGHES CM, PLOWMAN GD, SELDEN, C AND GULLICK WJ. (1992). Expression of the c-*erb*B-3 protein in normal human adult and fetal tissues. *Oncogene*, **7**, 1273–1278.

RAJKUMAR T, GOODEN CSR, LEMOINE NR AND GULLICK WJ. (1993). Expression of the c-*erb*B-3 protein in gastrointestinal tumours determined by monoclonal antibody RTJ1. *J. Pathol.*, **170,** 271-278.

SAINSBURY JRC, FARNDON JR, NEEDHAM, GK, MALCOLM AJ AND HARRIS AL. (1987). EGF-R status as a predictor of early recurrence of and death from breast cancer. *Lancet*, **ii,** 1398-1402.

SCAMBIA G, PANICI PB, BATTAGLIA F, FERRANDINA G, BAIOCCHI G, GREGGI S, DE VINCENZO R AND MANCUSO S. (1992). Significance of epidermal growth factor in advanced ovarian cancer. *J. Clin. Oncol.*, **10,** 529-535.

SLAMON DJ, GODOLPHIN W, JONES LA, HOLT JA, WONG SG, KEITCH DE, LEVIN WJ, STUART SG, UDOVE J, ULLRICH A AND PRESS MF. (1989). Studies of the HER-2/neu proto-oncogene in human breast and ovarian cancer. *Science*, **244,** 707-712.

VINDELOV LL, CHRISTENSEN IJ AND NISSEN NI. (1983). A detergent/trypsin method for the preparation of nuclei for flow cytometric analysis. *Cytometry*, **3,** 323-327.